# A large scale survey reveals that chromosomal copy-number alterations significantly affect gene modules involved in cancer initiation and progression

**DOI:** 10.1186/1755-8794-4-37

**Published:** 2011-05-06

**Authors:** Eva Alloza, Fátima Al-Shahrour, Juan C Cigudosa, Joaquín Dopazo

**Affiliations:** 1 Department of Bioinformatics and Genomics, Centro de Investigación Príncipe Felipe (CIPF), Valencia, Spain; 2 Broad Institute, 7 Cambridge Center, Cambridge, MA 02142, USA; 3 CIBER de Enfermedades Raras (CIBERER), ISCIII, CIPF, Valencia, Spain; 4 Molecular Cytogenetics Group. Centro Nacional de Investigaciones Oncologicas (CNIO), Madrid, Spain; 5 Functional Genomics Node (INB), CIPF, Valencia, Spain

## Abstract

**Background:**

Recent observations point towards the existence of a large number of neighborhoods composed of functionally-related gene modules that lie together in the genome. This local component in the distribution of the functionality across chromosomes is probably affecting the own chromosomal architecture by limiting the possibilities in which genes can be arranged and distributed across the genome. As a direct consequence of this fact it is therefore presumable that diseases such as cancer, harboring DNA copy number alterations (CNAs), will have a symptomatology strongly dependent on modules of functionally-related genes rather than on a unique "important" gene.

**Methods:**

We carried out a systematic analysis of more than 140,000 observations of CNAs in cancers and searched by enrichments in gene functional modules associated to high frequencies of loss or gains.

**Results:**

The analysis of CNAs in cancers clearly demonstrates the existence of a significant pattern of loss of gene modules functionally related to cancer initiation and progression along with the amplification of modules of genes related to unspecific defense against xenobiotics (probably chemotherapeutical agents). With the extension of this analysis to an Array-CGH dataset (glioblastomas) from The Cancer Genome Atlas we demonstrate the validity of this approach to investigate the functional impact of CNAs.

**Conclusions:**

The presented results indicate promising clinical and therapeutic implications. Our findings also directly point out to the necessity of adopting a function-centric, rather a gene-centric, view in the understanding of phenotypes or diseases harboring CNAs.

## Background

For many years, research into the genetic basis of many different diseases has cumulated a large corpus of knowledge on the links between particular genes and diseases. However, most diseases are multigenic and cannot be explained or characterized by a single gene but rather by a combination of genes [1]. In a broad sense, multigenecity reflects disruptions in proteins that participate in a protein complex, in a pathway [2] or, in general, in any known or yet to be discovered functional unit or module.

It is widely accepted that most of the biological functionality of the cell arises from complex interactions between their molecular components that define functional entities or modules that operate as small subcellular systems [3]. Recent evidence shows that genes mapping in close physical locations in the chromosomes tend to have a high degree of co-expression, that can often be linked to common functionality [4]. We have recently demonstrated that chromosomal regions enriched in certain types of functions have arisen at determinate evolutionary moments and have maintained since then in the genomes along the evolution [5]. It is a well established fact that chromosomal regions change their activity in diseases that harbor copy number alterations, such as some cancers [6]. Moreover, examples of disease-related genes mapping together in the genome have recently been described [7,8].

Despite these observations, the strategy followed in order to study pathologies harboring CNAs is still based on finding one or a few target genes [9]. However, it should be expected that modules composed of functionally-related genes, rather than one or a few key genes, were affected by the chromosomal alterations.

The aim of this study is to evaluate the importance of the functional component that can be attributed to the co-localization of modules of functionally related genes in those regions of the chromosomes that are lost or gained during the curse of the disease, and the possible impact that such component can have on its symptomatology. An interesting model to test this hypothesis is cancer. Genetic instability, producing not only point mutations but mainly CNAs, has long been recognized as a fundamental feature of many cancer types [10]. It has been suggested that only a few essential functional alterations in the physiology of the cells, the so-called hallmarks of cancer, are behind the vast catalog of cancer genotypes [11]. Our hypothesis is that in most cases such functions will be collectively carried out by modules of functionally-related genes that lay close together in the chromosomes. Such modules could consequently be found by analyzing the genomic regions most affected by chromosomal instability.

To this end, a functional analysis of the genes located in the regions undergoing copy number alterations in different cancer types has been conducted. This analysis aims to detect enrichments in cancer-related functions significantly associated to chromosomal regions that are systematically gained or lost in cancers. The conventional way to determine the level of involvement of a gene in the disease would imply the establishment of a threshold beyond which the participation of the gene in deletions of amplifications can be declared significant. The way in which this threshold is fixed tends to be arbitrary and can compromise the conclusions of the work [12]. To avoid the imposition of artificial thresholds, we have applied a generalization of the concept of gene set analysis (GSA), previously proposed in the context of transcriptomics [13] and further generalized to other genomic domains such as evolution [14], QTL analysis [15] and genotyping [16]. In the approach used here a GSA test [14] is conducted to relate pre-defined sets of functionally-related genes to a variable that represents the propensity of a gene to be involved in a deletion or in an amplification. Such variable is the absolute frequency at which each gene is affected by copy number alterations (CNAs) because it is located in a lost or gained chromosomal region. This family of methods based on the analysis of functionally-related gene sets, (modules therein), are known to be more sensitive than other alternative functional enrichment methods [17-19].

Array-CGH constitute an ideal type of data, given that they provide a detailed and accurate description of the regions gained and lost and consequently a well defined characterization of the genes included in them. Unfortunately, there are still a limited number of such arrays available in the databases. For example the progenetix database [20], a repository of cytogenetic abnormalities in cancer, contains only 3016 samples of array-CGH at present (August 2009). The more general ArrayExpress database contains 34 experiments, only 10 of which contain the word cancer, summing up a total of only 463 samples. Both databases are highly overlapping and include data from different platforms which makes the analysis even more difficult [21]. With the exception of initiatives such as The Cancer Genome Atlas [22], in which large numbers of cancers are simultaneously analyzed with Array-CGH, most experiments involve small numbers of samples. Small sample sizes of many experiments hinder the possibility of obtaining significant associations between gene modules and regions gained or lost in cancers. However, this situation will eventually change in the near future.

On the other hand, classical CGH assays provide an alternative low-resolution description of regions gained or lost. Fortunately, a large number of such observations is available in repositories such as the Mitelman database [23]. This is a manually curated repository that relates chromosomal aberrations to tumour features, based information extracted from the literature. Here we conducted a systematic analysis of more than 140.000 observations of CNAs corresponding to losses and gains of genetic material in cancers. The results revealed a significant pattern of losses and gains of modules of functionally-related genes that will affect physiological processes associated to cancer initiation and progression. Consequently, a function-centric view, which takes into account the cooperative effects of the genes that integrate functional modules, can provide a more complete understanding of diseases that harbor CNAs.

## Results

### Distribution of CNAs

Classical cytogenetic techniques are more suitable for detecting regional deletions (deletions affecting to regions of the chromosomes, accounting for 19859 out of a total of 86048 deletions reported in the Mitelman database) than for detecting regional amplifications (only 1011 out of a total of 55935) so, there is an unbalance between the number of observations in each case, which constitutes an obvious limitation in the conclusions obtained for amplifications using this type of data. Apparently this limitation did not seem to apply to whole chromosome CNAs. There was a clear significant negative correlation between the chromosome size and the number of observed whole chromosome CNA deletions. Probably this observation is reflecting the obvious general fact that the smaller chromosomes have fewer genes and their loss is potentially less damaging for the cell. On the other hand, no equivalent trend could be observed in the case of whole chromosome gains. In this case an average of ~2000 observations (SD ~1000) was recorded for all the chromosomes, except 7, 8 and 21, with 4156, 7013 and 4974 observations respectively. These relevant exceptions were due to the specific and highly recurrent presence of these chromosome aneuploidies in well defined epithelial and hematological cancers.

Particular behaviors of some chromosomes deserve to be mentioned. For example, chromosome Y displayed a large number of deletions but an abnormally low number of amplifications. An opposite trend was observed for chromosomes 8 and 21. Particularly interesting is chromosome 7 that showed an unexpectedly high number of amplifications and deletions simultaneously.

Although these observations have been extensively reported, nothing is known about the advantageous biological reasons that a particular tumor may have due to the acquisition of a chromosomal aberration along its evolution.

### Functional roles found in frequently lost regions

**Figure 1:**
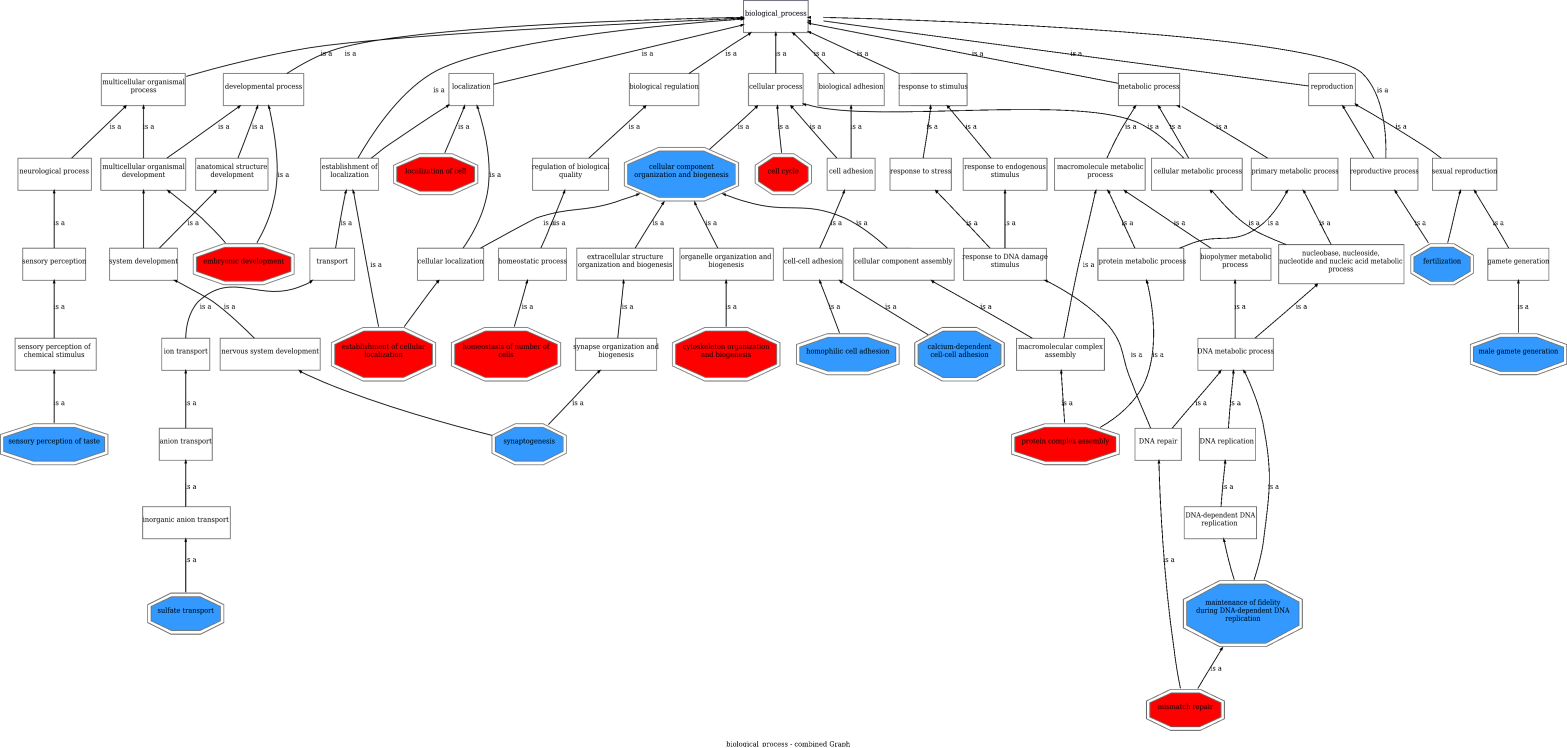
**GO terms significantly associated to chromosomal regions frequently lost in cancers**. Since a large number of GO terms are tested, the popular FDR method [34] was used to adjust the p-values for multiple-testing effects obtained when conducting a one-tailed test [14]. GO terms with p-values under a conventional threshold of p < 0.05 were represented as red octagons and GO terms under a more liberal 0.05 < p < 0.15 were represented as blue octagons. White squares represent non-significant terms connecting the significant terms found. The picture has been obtained using the GoGraphViewer utility of the Babelomics package http://www.babelomics.org[44].

The analysis of 86048 deletion cases revealed a significant loss of gene modules, defined by GO terms, that can be related to the acquisition of most of the cancer hallmarks [11]. The possible roles in cancer of the GO terms found can be found by using text-mining tools, such as the GOPubMed server [24]. Thus, mutability could be increased in cancer cells by the loss of gene modules with GO definitions such as "maintenance of fidelity during DNA-dependent DNA replication" (p = 0.0299) and its direct descendant in the GO hierarchy "mismatch repair" (p = 0.0942). "Cytoskeleton organization and biogenesis" (p = 0.1488) has been linked to genetic instability [25]. Metastatic state and tissue invasion could be acquired by cancer cells when modules such as "homophilic cell adhesion" (p = 3.8779 × 10^-17^), and "calcium-dependent cell adhesion" (p = 2.0064 × 10^-08^), both descendants of "cell adhesion" (see Figure 1) are lost. Another module, whose loss is probably involved in the metastatic state and tissue invasion too, is "Localization of cell" (p = 0.0651), that refers to processes by which a cell is transported to, and/or maintained in, a specific location, related to the transport and maintenance of proteins in the cell membrane. The ontogenic relationships among GO terms depicted in Figure 1 allows tracking "sulfate transport" (p = 0.0282) gene modules to processes related to "establishment of localization", a parent term of "establishment of cellular localization" and putatively involve them in tissue invasion and metastasis. Modules involved in "homeostasis of number of cells" (p = 0.1265) are also lost, probably contributing to the self-sufficiency in growth signals and insensitivity to antigrowth signals, two well known properties of cancer cells. The loss of "interferon-alpha/beta receptor binding" (p = 0.0223) function is probably mimicking the down-regulation of β interferon (a factor which is known to elicit angiogenesis [26]) at cellular level. It is also known that extracellular proteases are functionally linked to pro-angiogenic integrins and both promote the invasive potential of angiogenic endothelial cells [27]. The loss of "serine-type endopeptidase inhibitor activity" (p = 0.0061) could be behind the increase of such proteases. Table 1 and Additional file 1 Tables S1, S2 and S3 contain modules involved in other enzymatic functions related to cell adhesion processes. Modules of genes located in cell membranes and in intracellular junctions have been significantly lost (Table 1) as well. Interestingly, gene modules of cytoskeleton filaments or microtubules were also significantly lost. It is well known that the loss of genes in these cellular structures leads to chromosomal instability [25]. Since chromosomal instability generates genetic heterogeneity, and it is fundamental to the processes of neoplastic progression, it has recently been proposed as another hallmark of cancer [28]. Some features marked as "unrelated" in Table 1 are not directly related to cancer and most probably account for specialized characteristic of the differentiated cells that originated different cancer types. Some of these features might have been discarded in the process of indifferentiation process followed by many cancers.

**Table 1:** GO terms most significantly associated to chromosomal regions frequently lost in cancers.

GO term	GO ID	Number of genes	p-value (FDR-adjusted)	p-value (Bonferroni-adjusted)	Cancer feature
**Biological process**					

homophilic cell adhesión	GO:0007156	114	3.8779 × 10^-17^	4.77 × 10-^18^	Metastasis and invasion

calcium-dependent cell-cell adhesion	GO:0016339	22	2.0064 × 10^-08^	9.88 × 10^-9^	Metastasis and invasion

synaptogenesis	GO:0007416	30	1.2491 × 10^-05^	1.46 × 10^-6^	Unrelated

sensory perception of taste	GO:0050909	18	0.00510791	0.00299	Unrelated

cellular component organization and biogenesis	GO:0016043	2165	0.01731139	0.0764	Replicative potential

fertilization (sensu Metazoa)	GO:0009566	54	0.02135039	0.0648	Unrelated

sulfate transport	GO:0008272	12	0.02815271	0.00961	Metastasis and invasion

maintenance of fidelity during DNA-dependent DNA replication	GO:0045005	28	0.02990008	0.0141	Mutability

male gamete generation	GO:0048232	210	0.0449652	0.210	Unrelated

localization of cell	GO:0051674	410	0.06513653	0.697	Metastasis and invasion

mismatch repair	GO:0006298	27	0.09421995	0.0113	Mutability

cell cycle	GO:0007049	805	0.09599886	1.00	Replicative potential

**Molecular function**					

pancreatic ribonuclease activity	GO:0004522	15	0.0046537	0.000623	Metastasis and invasion?

serine-type endopeptidase inhibitor activity	GO:0004867	89	0.0060532	0.000767	Angiogenesis

nucleotide binding	GO:0000166	1993	0.0064466	0.00183	Mutability?

sulfate porter activity	GO:0008271	10	0.0078682	0.00279	Metastasis and invasion

interferon-alpha/beta receptor binding	GO:0005132	10	0.022324	0.0180	Angiogenesis

carboxypeptidase A activity	GO:0004182	25	0.032232	0.0156	Metastasis and invasion?

lipoxygenase activity	GO:0016165	7	0.040851	0.0696	Metastasis and invasion?

**Cellular component**					

cytoskeletal part	GO:0044430	613	0.0000647	0.0000153	Chromosomal instability

microtubule organizing center part	GO:0044450	26	0.014677	0.00849	Chromosomal instability

intermediate filament cytoskeleton	GO:0045111	153	0.014677	0.00924	Chromosomal instability

integral to plasma membrane	GO:0005887	1180	0.042474	1.50 × 10-001	Metastasis and invasion

Given that many GO terms are tested, the popular FDR and Bonferroni methods were used to adjust for multiple-testing effects the p-values obtained when conducting a one-tailed test (see details in [14]). GO terms with p-values under a conventional threshold of p < 0.05 are listed. GO terms under a more liberal 0.05 < p < 0.15 are listed in Additional file 1 Tables S1, S2 and S3. Rightmost column makes reference to the cancer feature most probably related to the GO term.

All the functions described have been obtained by analyzing all the cancers together. It is difficult to particularize the analysis in individual cancers given the poor resolution of the technology and the comparatively fewer cases recorded for individual cancers. Only in the case of leukemia, with 6859 cases available, was it possible to find significant terms (see Additional file 1 Table S4). Actually, a richer and more detailed picture of the functions of the modules affected by the deletions is obtained. Thus, in addition to processes related to cell adhesion, localization of cell, sulfate transport and other processes described above, more specific terms for signaling were found, such as "cell-cell signaling" (p = 5.79 × 10^-03^) and some descendants or "cell surface receptor linked signal transduction" (p = 5.18 × 10^-03^). Other interesting terms that can be associated to metastasis would be "regulation of cell migration" (p = 0.0269). Distinct modules related to phosporylation processes have also been significantly lost. Finally, processes related to the immune system, probably characteristic of this type of cancer, were also lost, such as "antigen processing and presentation" (p = 0.0159).

Additional File 2 contains the list of the genes for each significant GO term with the corresponding chromosomal locations and the number of CNAs in which they are involved.

### Functional roles found in frequently amplified regions

A similar analysis was performed with amplifications although it turned out to be less conclusive because there were far fewer cases corresponding to regional amplifications (only 1011), probably because such amplifications are more difficult to detect and properly characterize with conventional cytogenetic analysis. Most of the data used came from observations of whole-chromosome amplifications (54924 cases), so a higher imprecision in the definition of the functional gene modules is expected in this type of data. Table 2 shows biological processes significantly associated to regions frequently amplified in human cancers. The GO terms found refer to very general processes, so the conclusions for the amplifications are necessarily more speculative. Most of the processes significantly amplified by cancer cells are related to unspecific defense responses. All of them, but especially "xenobiotic metabolic process" (p = 0.028646), are most probably behind the response of the cancer cells against the chemotherapy. Apart from the unspecific defense character of the processes "defense response to fungus" (p = 0.018143) and "defense response to bacterium" (p = 1.759 × 10^-11^) both are significantly over-represented in chromosome 8 (see next section), which is one of the most frequently amplified. The biological process "telomere maintenance" (with p = 0.14336, a marginal significance if we consider a liberal threshold of 0.15) is quite general but clearly accounts for the immortalization of the cancer cells. Nevertheless we cannot conclude whether the term "mismatch repair" (p = 0.003339) has been amplified because it represents gene modules related to any child term such as "negative regulation of mismatch repair" or because it is present in chromosome 7 which exhibits a high number of deletions and amplifications simultaneously. In any case, if chromosome 7 is examined for functional enrichment [29] the term "mismatch repair" is significantly over-represented in it.

**Table 2:** GO terms corresponding to the "biological process" ontology significantly associated to chromosomal regions frequently amplified in cancers.

GO term	GO ID	Number of genes	p-value (FDR-adjusted)	p-value (Bonferroni-adjusted)
defense response to bacterium	GO:0042742	**98**	**1.7594 × 10^-11^**	**2.98 × 10^-11^**

mismatch repair	GO:0006298	**27**	**0.003339**	**0.0020**

xenobiotic metabolic process	GO:0006805	**29**	**0.028646**	0.169

defense response to fungus	GO:0050832	**13**	**0.018143**	0.101

telomere maintenance	GO:0000723	24	0.14336	0.303

GO terms with p-values under a conventional threshold of p < 0.05 are listed in boldface. Also, GO terms under a more liberal p < 0.15 are listed in normal face. Nominal p-values obtained by conducting the one-tailed test (see details in [14]) were adjusted for multiple testing using the FDR [34] and the more conservative Bonferroni corrections.

### Chromosome-specific functional roles

While many of the observations made for functional roles lost are based on regional deletions, the observations made for functional roles gained are almost exclusively based on whole chromosome gains. This fact suggests the existence of a strong regionalization of the functional units composed by gene modules. While the concentration of functionally-related genes in neighborhoods has already been described [4], the existence of chromosome-specific functions, beyond the obvious ascribed to the sexual chromosomes, has not systematically been explored. Additional file 1 Table S5 shows the results obtained upon the application of a functional enrichment method [29] that finds significant over-representations of functional terms in each chromosome. Surprisingly only a few chromosomes -12, 13, 14, 15 and 21- are not significantly enriched in any functionally-related gene module. Some of the chromosome-specific gene modules present in frequently amplified chromosomes correspond, obviously, to functions systematically gained in cancers (e.g. defense response to bacterium or defense response to fungus in chromosome 8 or mismatch repair in chromosome 7). Although the existence of biological functions specific of one or of a few chromosomes does not seem to be a frequent occurrence (we must remember that there are several thousands of GO terms), some of these, however, are clearly over-represented in some chromosomes. This observation suggests that when a complete chromosome is lost or gained (a frequent event in cancer) the activity of whole modules of genes carrying out a particular biological role are dramatically changed.

### Fine-scale analysis of CNAs of functional roles lost in glioblastoma

**Figure 2:**
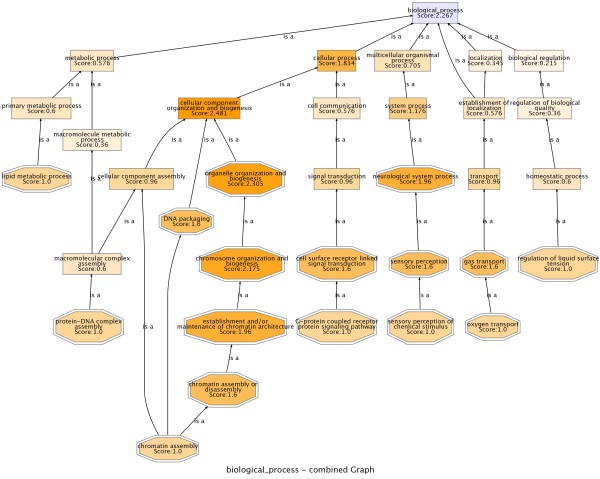
**GO terms significantly associated to chromosomal regions frequently lost in glioblastomas **[30]. Since a large number of GO terms are tested, the popular FDR method [34] was used to adjust the p-values for multiple-testing effects obtained when conducting a one-tailed test [14]. GO terms with p < 0.05 were represented as octagons. White squares represent non-significant terms connecting the significant terms found. The picture has been obtained using the GoGraphViewer utility of the Babelomics package http://www.babelomics.org[44].

After proving the sensitivity of the method proposed for analyzing the functional impact of CNAs in a large dataset with low resolution we essayed a smaller dataset with higher resolution. We downloaded 294 Array-CGH experiments of glioblastomas [30] from The Cancer Genome Atlas [22]. The data was already segmented in the repository and the CNA coordinates were available and well defined. Despite the fact that the number of samples is comparatively smaller than in the case of CGH (in the range of one order of magnitude lower) the resolution of the Array-CGH was good enough to obtain significant results. Table 3 lists the functional modules present in regions significantly lost. The losses of several processes are relevant in the particular context of this cancer. For instance, the highly significant "neurological process" (p = 4.45 × 10^-12^) and their descendants (see Figure 2) are probably related to the undifferentiation process o neuronal cells. Also other processes related to chromosomal instability are related, such as "chromatin assembly" (p = 1.60 × 10^-05^) and a number of parent terms have been lost. Several descendants of "signal transduction", probably related to self-sufficiency in growth signals, have also been significantly lost. Interestingly, "oxygen transport" (p = 0.0263), was also found to be significantly lost. Our group recently proved the link between undifferentiation and hypoxia [31]. It is tempting to speculate that the loss of genes related to oxygen transport can be used by the cancer to gain or to mimic some sort of state of undifferentiation.

**Table 3:** GO terms corresponding to the "biological process" ontology significantly associated to chromosomal regions frequently lost in the glioblastomas [30].

GO term	GO ID	p-value (FDR-adjusted)	p-value (Bonferroni-adjusted)
sensory perception of chemical stimulus	GO:0007606	9.83 × 10^-24^	1.97 × 10^-17^

sensory perception	GO:0007600	2.39 × 10^-17^	7.17 × 10^-14^

neurological process	GO:0050877	4.45 × 10^-12^	1.78 × 10^-06^

G-protein coupled receptor protein signaling pathway	GO:0007186	5.44 × 10^-8^	0.0272

chromatin assembly	GO:0031497	1.60 × 10^-5^	0.000799

chromatin assembly or disassembly	GO:0006333	1.85 × 10^-5^	0.00011

cell surface receptor linked signal transduction	GO:0007166	0.00028	0.00169

DNA packaging	GO:0006323	0.00204	0.01740

establishment and/or maintenance of chromatin architecture	GO:0006325	0.00204	0.02042

protein-DNA complex assembly	GO:0065004	0.00204	0.01926

chromosome organization and biogenesis (sensu Eukaryota)	GO:0007001	0.00217	0.02389

chromosome organization and biogenesis	GO:0051276	0.00234	0.02819

lipid metabolic process	GO:0006629	0.02525	0.17675

gas transport	GO:0015669	0.02628	0.15771

oxygen transport	GO:0015671	0.02628	0.15771

organelle organization and biogenesis	GO:0006996	0.03449	0.41390

regulation of liquid surface tension	GO:0050828	0.04568	0.04568

The one-tailed test, as implemented in the GSA version provided by the Babelomics package [44], was used (see details in [14]). Nominal p-values were adjusted for multiple testing using the FDR [34] and the Bonferroni methods.

The results demonstrate the importance of having high-resolution Array-CGH data. With only 294 samples we have found well defined and highly significant terms. In the conventional CGH case we needed almost two orders of magnitude more samples to obtain similar results.

## Discussion

Given the poor resolution that classical cytogenetic techniques have, the analysis presented here can be considered as a coarse-grain view of the real importance of neighborhoods of modules composed by functionally-related genes. The extension of this methodology to the analysis of the data produced by Array-CGH [32] has demonstrated to be straightforward. Also the results are noticeably better when a reasonable number of samples are analyzed. The forthcoming deluge of data foreseeable from such high-throughput platforms will allow refining the results presented here in a near future. Moreover, more sophisticated methods of error correction in the GSA methods will result in more specific and accurate findings in the future [33]. In this study we have used the popular FDR, that keeps false discoveries at a reasonable rate without being too conservative [34].

Despite its low resolution and limitations, the analysis based on cytogenetic data resulted to be sensitive enough to reveal the existence of an important number of gene modules, significantly associated to regions systematically deleted in cancers, whose functionalities (as represented by GO terms) clearly account for cancer-related biological processes and molecular roles. Similarly, regions systematically amplified by cancer cells are significantly populated by genes carrying out processes related to cell immortalization and to unspecific defense (probably against chemotherapeutical agents). When the vast catalog of cancer cell genotypes is analyzed in detail, a small number of functional alterations, also denominated hallmarks of cancer by some authors [11,28], seems to account for all this complexity. These altered functions shared by most (if not all) cancers are: self-sufficiency in growth signals, insensitivity to antigrowth signals, evasion of apoptosis, limitless replicative potential, sustained angiogenesis, and tissue invasion and metastasis [11]. Interestingly, losses and gains of gene modules carrying out functions (whose GO terms are compatible with the acquisition of all of them) were found. Moreover, another well-established feature of cancer, the acquisition of multi drug resistance by amplification of chromosomal regions [35] was also found to depend on the amplification of the corresponding gene modules (amplification of the "xenobiotic metabolic process" GO term; see table 2). Finally, the enabling characteristic for the acquisition of these functionalities, genomic instability, also considered a cancel hallmark by other authors [28], has been clearly detected by our analysis. The analysis of leukemia in the CGH data and the analysis of the TCGA glioblastomas data [30] revealed, in addition to processes related to general cancer properties, other affected processes related to particular characteristics of the cancer types.

The emerging picture from this study is a scenario in which cancer cell populations undergo a Darwinian dynamics along the distinct phases of the disease [36], which mainly occurs by genetic modifications caused by chromosomal instability [10,28]. This chromosomal instability, affecting large chromosomal regions, is the substrate of a strong selective pressure operating on a reduced number of cellular functions leading to cancer [37]. Our hypothesis, consistent with the results found, is that in a considerable number of cases such functions will be carried out by modules of genes that are located in neighborhoods along the chromosomes rather than by one or few key genes.

Regardless of the fact that some recent evidences link chromosomal physical location to function [4,5] and that disease-related genes have been recently described as mapping in adjacent positions in the chromosomes [6-8], the putative impact that this local distribution of cellular functionality could have in the symptomatology of diseases that harbor CNAs still remains largely unexplored.

## Conclusions

The systematic study presented here has firmly related, for the first time, molecular functional roles of gene modules involved in CNAs to biological processes related to cancer initiation and progression. Our conclusions point to the necessity of taking a more function-centric perspective to understand the functional effect of CNAs, especially in the context of cancer. Even more important is the relevance of these findings for the design of therapeutic strategies in the treatment of cancer. Also, drug discovery processes need to account for this new scenario [38,39]. The recent observation of an unexpectedly widespread distribution and prevalence of CNA polymorphisms in the human genome [32] makes even more urgent the adoption of a viewpoint in which gene modules, instead of genes in isolation, are considered to be behind most phenotypes or disease symptoms. Our findings are consistent with the idea that pathways, rather than individual genes, appear to govern the course of tumorigenesis [40,41].

## Methods

### Database of chromosomal alterations

The Mitelman database of Chromosome Aberrations in Cancer http://cgap.nci.nih.gov/Chromosomes/Mitelman was used as primary source of information. A total of 86048 observations (independent CNA event described in the database) of deletions (19859 of them corresponding to regional deletions affecting only to parts of the chromosomes and 66189 to whole chromosome deletions), including any type of cancer, were obtained from the database. Each individual case in the database has a description of the associated CNA in a standard format with the indication of the cytoband or cytobands affected by the deletion or the amplification; for example del(7)(p13p15). Although with a smaller coverage, amplifications were also stored in the database. A total of 55935 observations of amplifications corresponding to 1011 regional amplifications and 54924 whole chromosome amplifications were retrieved from the database.

In order to avoid possible artifacts of erroneous observations, we have only taken into account deletions or amplifications occurring at least in two cases.

### Array-CGH data from TCGA

The Cancer Genome Atlas [22] was used as the source for Array-CGH data on cancer. A large experiment with 294 Array-CGH (Agilent 244 K) of glioblastoma was used [30]. The data already segmented was available and was retrieved for the analysis.

### Gene mapping

Genes were mapped to their corresponding cytobands using the Ensembl database [42], release 45, Homo sapiens (NCBI 36).

### Functional annotations and representation

The widely accepted GO functional categories [43] were used for the functional profiling of the genes involved in CNAs. The GO annotations for the human genes were provided by the ID-converter engine of the Babelomics package [44]http://www.babelomics.org which contains annotations taken from the GO database [43]. GO terms corresponding to levels 3 to 6 in the hierarchy were tested. This correspond to a total of 3549 Biological Process, 2817 Molecular Function and 394 Cellular Component GO terms

Plots of the relationships existent among GO terms can be obtained by using the GO visualization tools implemented in the Babelomics package [44].

### Chromosomal functional enrichment

The significance in the enrichment of functional terms in chromosomes was carried out with the FatiGO [29] program as implemented in the Babelomics package [44]http://www.babelomics.org. Briefly, the program builds a 2 × 2 contingency table for each functional term checked and applies an exact Fisher's test. The resulting p-values are adjusted to compensate multiple-testing effects using the popular FDR method [34].

### Functional profiling of genes more frequently involved in CNAs

**Figure 3:**
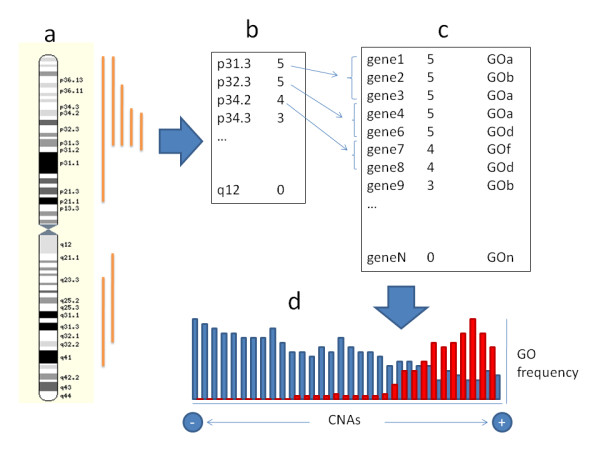
**Schematic representation of the gene-set enrichment procedure followed**. (**a**) The CNAs are mapped onto the chromosomal coordinates and then used to (**b**) build up a ranked list of cytobands, which is further used to (**c**) define a ranking of genes according to how frequently are they involved in CNAs. The distribution of gene modules, in this case the annotations of a fictitious GO, derived from such ranked list is tested for its significant accumulation in frequently lost regions. (**d**) The variant of the GSA test used [14] seeks for significant asymmetrical distributions of annotations (red bars) with respect to the annotation background (blue bars). The × axis in **d **represents the number of cases in which a gene has been observed in region affected by a CAN (that, generally speaking can be either amplification or a loss). The bar height represents the number of genes involved in a given frequency of CNA events. The red bars represent the distribution of the genes of a given GO across the frequency of observations of CNAs. The GSA test seeks for GOs whose distributions are significantly skewed towards high or low frequencies of CNAs. Since a large number of GO terms are tested, the FDR method [34] was used to adjust the p-values for multiple-testing effects. The Mitelman database of Chromosome Aberrations in Cancer http://cgap.nci.nih.gov/Chromosomes/Mitelman, May 2007 release, was used as primary source of information. A total of 86048 observations of deletions (corresponding 19859 to regional deletions and 66189 to whole chromosome deletions), and 55935 observations of amplifications (corresponding to 1011 regional amplifications and 54924 whole chromosome amplifications) including any type of cancer, were obtained from the database.

Since the annotation, given the resolution of the cytogenetic technique, was made at the level of cytobands, these were chosen as operational units for this study. Different deletions in different cases spanned over distinct chromosomal regions (Figure 3a). Then, the number of times that a cytoband was deleted or was part of a larger deletion was counted up. Next, the cytobands were ranked according to the frequency of its occurrence in the observations reported in the database (Figure 3b). These frequency values were used to build up a list of genes ranked according to the number of times the cytoband in which they were located was lost in the observations (Figure 3c). Once this list is completed, the association of modules of functionally-related genes (defined here using GO terms) to high values of the rank (that is: high frequency of deletion; see Figure 3d) is tested. Exactly the same process can be followed with amplifications.

We used for this purpose a gene-set functional profiling method. This methodology, introduced in the field of microarray data analysis [13], comprises a family of methods that allows studying the coordinate over- or under-representation of pre-defined gene sets in the extremes of a list of genes ranked by some criteria [45]. In particular we have used a generalized GSA test [14] implemented in the Babelomics package [44]. This algorithm has the advantage of dealing directly with the ranked list, without requiring either any extra information or the original experimental values [14]. In particular, the test seeks for significant asymmetrical distributions of GO terms across consecutive partitions of the ranked list [14]. If the term is systematically over-represented in the upper part of the partitions (corresponding to the genes more frequently deleted) we will consider that this GO functionality is systematically deleted. The same rationale can be applied to the list ranked by frequency of amplifications. The FDR method [34] was used to adjust the p-values for multiple-testing effects given that a large number of GO terms are tested.

GO definitions [43], obtained as explained above, were used to define such gene modules composed of functionally-related genes.

### Validation of the roles of biological processes in cancer

The GoPubmed [24], a tool that relate terms to genes using sophisticated textmining methods was used to check whether the biological processes found were linked to cancer by co-citations in the scientific literature.

## Competing interests

The authors declare that they have no competing interests.

## Authors' contributions

EA carried out the analysis of the data. FA-S contributed with the software for the enrichment analysis. JCC wrote the biological pat of the results and discussion. JD conceived and coordinated the work and wrote the paper. All the authors have read and approved the final manuscript.

## Pre-publication history

The pre-publication history for this paper can be accessed here:

http://www.biomedcentral.com/1755-8794/4/37/prepub

## Supplementary Material

Additional file 1 **File containing supplementary tables**. Contains the following additional tables: Additional Table S1. GO terms corresponding to the "biological process" ontology significantly associated to chromosomal regions frequently lost in cancers. The one-tailed test, as implemented in the GSA version provided by the Babelomics package was used. Nominal p-values were adjusted for multiple testing using the FDR. Liberal p-values < 0.15 are listed in the table. P-values < 0.05 are represented in boldface. GO terms are arranged by p-values. See Figure 1 for a representation of the relationships among he GO terms. Additional Table S2. GO terms corresponding to the "molecular function" ontology significantly associated to chromosomal regions frequently lost in cancers. The one-tailed test, as implemented in the GSA version provided by the Babelomics package was used. Nominal p-values were adjusted for multiple testing using the FDR. Liberal p-values < 0.15 are listed in the table. P-values < 0.05 are represented in boldface. GO terms are arranged by p-values. Additional Table S3. GO terms corresponding to the "cellular component" ontology significantly associated to chromosomal regions frequently lost in cancers. The one-tailed test, as implemented in the GSA version provided by the Babelomics package was used. Nominal p-values were adjusted for multiple testing using the FDR. Liberal p-values < 0.15 are listed in the table. P-values < 0.05 are represented in boldface. GO terms are arranged by p-values. Additional Table S4. GO terms corresponding to the "biological process" ontology significantly associated to chromosomal regions frequently lost in leukemia. The one-tailed test, as implemented in the GSA version provided by the Babelomics package was used. Nominal p-values were adjusted for multiple testing using the FDR. Liberal p-values < 0.15 are listed in the table. P-values < 0.05 are represented in boldface. GO terms are arranged by p-values. Additional Table S5. GO terms corresponding to the "biological process" ontology significantly over-represented in the chromosomes found by the functional enrichment test implemented in the FatiGO program. The one-tailed test, as implemented in the program was used to check for significance. Nominal p-values were adjusted for multiple testing using the FDR. GO terms are arranged by p-values. Click here for file

Additional file 2 **ZIP file containing tab-delimited text files for GO terms**. One text file for each significant GO term. Each file contains one line per gene belonging to the GO. Each line lists the Ensembl ID, the Gene Name, the number of cases in which the gene was involved in a CNA, and its location including: Chromosome, Band, Start (bp) and End (bp). Click here for file
