# Supplementary material for: A large scale survey reveals that chromosomal copy-number alterations significantly affect gene modules involved in cancer initiation and progression
Source: BMC Med Genomics. 2011 May 6;4:37. doi: 10.1186/1755-8794-4-37 (PMC3112060; doi:10.1186/1755-8794-4-37)
Supplement: Additional file 1 — File containing supplementary tables. Contains the following additional tables: Additional Table S1. GO terms corresponding to the "biological process" ontology significantly associated to chromosomal regions frequently lost in cancers. The one-tailed test, as implemented in the GSA version provided by the Babelomics package was used. Nominal p-values were adjusted for multiple testing using the FDR. Liberal p-values < 0.15 are listed in the table. P-values < 0.05 are represented in boldface. GO terms are arranged by p-values. See Figure 1 for a representation of the relationships among he GO terms. Additional Table S2. GO terms corresponding to the "molecular function" ontology significantly associated to chromosomal regions frequently lost in cancers. The one-tailed test, as implemented in the GSA version provided by the Babelomics package was used. Nominal p-values were adjusted for multiple testing using the FDR. Liberal p-values < 0.15 are listed in the table. P-values < 0.05 are represented in boldface. GO terms are arranged by p-values. Additional Table S3. GO terms corresponding to the "cellular component" ontology significantly associated to chromosomal regions frequently lost in cancers. The one-tailed test, as implemented in the GSA version provided by the Babelomics package was used. Nominal p-values were adjusted for multiple testing using the FDR. Liberal p-values < 0.15 are listed in the table. P-values < 0.05 are represented in boldface. GO terms are arranged by p-values. Additional Table S4. GO terms corresponding to the "biological process" ontology significantly associated to chromosomal regions frequently lost in leukemia. The one-tailed test, as implemented in the GSA version provided by the Babelomics package was used. Nominal p-values were adjusted for multiple testing using the FDR. Liberal p-values < 0.15 are listed in the table. P-values < 0.05 are represented in boldface. GO terms are arranged by p-values. Additional Table S5. GO terms cor [file 1755-8794-4-37-S1.DOC]

# Additional Tables.

## A large scale survey reveals that chromosomal copy-number alterations significantly affect gene modules involved in cancer initiation and progression.

**Eva Alloza, Fátima Al-Shahrour, Juan Cruz Cigudosa, Joaquín Dopazo**

**Additional Table 1**. GO terms corresponding to the “biological process” ontology significantly associated to chromosomal regions frequently lost in cancers. The one-tailed test, as implemented in the GSA version provided by the Babelomics package [1] was used (see details in [2]). Nominal p-values were adjusted for multiple testing using the FDR [3]. Liberal p-values < 0.15 are listed in the table. P-values < 0.05 are represented in boldface. GO terms are arranged by p-values. See Figure 1 for a representation of the relationships among he GO terms.

| GO term | GO ID | p-value  (FDR-adjusted) |
| --- | --- | --- |
| homophilic cell adhesion | GO:0007156 | **3.8779x10^-17^** |
| calcium-dependent cell-cell adhesion | GO:0016339 | **2.0064x10^-08^** |
| synaptogenesis | GO:0007416 | **1.2491x10^-05^** |
| sensory perception of taste | GO:0050909 | **0.00510791** |
| cellular component organization and biogenesis | GO:0016043 | **0.01731139** |
| fertilization (sensu Metazoa) | GO:0009566 | **0.02135039** |
| sulfate transport | GO:0008272 | **0.02815271** |
| maintenance of fidelity during DNA-dependent DNA replication | GO:0045005 | **0.02990008** |
| male gamete generation | GO:0048232 | **0.0449652** |
| localization of cell | GO:0051674 | 0.06513653 |
| mismatch repair | GO:0006298 | 0.09421995 |
| cell cycle | GO:0007049 | 0.09599886 |
| homeostasis of number of cells | GO:0048872 | 0.1265005 |
| embryonic development | GO:0009790 | 0.14116797 |
| establishment of cellular localization | GO:0051649 | 0.14503085 |
| protein complex assembly | GO:0006461 | 0.14811236 |
| cytoskeleton organization and biogenesis | GO:0007010 | 0.14878051 |

**Additional Table 2.** GO terms corresponding to the “molecular function” ontology significantly associated to chromosomal regions frequently lost in cancers. The one-tailed test, as implemented in the GSA version provided by the Babelomics package [1] was used (see details in [2]). Nominal p-values were adjusted for multiple testing using the FDR [3]. Liberal p-values < 0.15 are listed in the table. P-values < 0.05 are represented in boldface. GO terms are arranged by p-values.

| GO term | GO ID | p-value  (FDR-adjusted) |
| --- | --- | --- |
| pancreatic ribonuclease activity | GO:0004522 | **0.0046537** |
| serine-type endopeptidase inhibitor activity | GO:0004867 | **0.0060532** |
| nucleotide binding | GO:0000166 | **0.0064466** |
| sulfate porter activity | GO:0008271 | **0.0078682** |
| interferon-alpha/beta receptor binding | GO:0005132 | **0.022324** |
| carboxypeptidase A activity | GO:0004182 | **0.032232** |
| lipoxygenase activity | GO:0016165 | **0.040851** |
| arylsulfatase activity | GO:0004065 | 0.080568 |
| gamma-glutamyltransferase activity | GO:0003840 | 0.081503 |
| taste receptor activity | GO:0008527 | 0.11090 |
| arylesterase activity | GO:0004064 | 0.11833 |
| hexosaminidase activity | GO:0015929 | 0.11833 |

**Additional Table 3.** GO terms corresponding to the “cellular component” ontology significantly associated to chromosomal regions frequently lost in cancers. The one-tailed test, as implemented in the GSA version provided by the Babelomics package [1] was used (see details in [2]). Nominal p-values were adjusted for multiple testing using the FDR [3]. Liberal p-values < 0.15 are listed in the table. P-values < 0.05 are represented in boldface. GO terms are arranged by p-values.

| GO term | GO ID | p-value  (FDR-adjusted) |
| --- | --- | --- |
| cytoskeletal part | GO:0044430 | **0.0000647** |
| microtubule organizing center part | GO:0044450 | **0.014677** |
| intermediate filament cytoskeleton | GO:0045111 | **0.014677** |
| integral to plasma membrane | GO:0005887 | **0.042474** |
| caveolar membrane | GO:0016599 | 0.055035 |
| perinuclear region | GO:0048471 | 0.077321 |
| intercellular junction | GO:0005911 | 0.079056 |

**Additional Table 4.** GO terms corresponding to the “biological process” ontology significantly associated to chromosomal regions frequently lost in leukemia. The one-tailed test, as implemented in the GSA version provided by the Babelomics package [1] was used (see details in [2]). Nominal p-values were adjusted for multiple testing using the FDR [3]. Liberal p-values < 0.15 are listed in the table. P-values < 0.05 are represented in boldface. GO terms are arranged by p-values.

| GO term | GO ID | p-value  (FDR-adjusted) |
| --- | --- | --- |
| homophilic cell adhesion | GO:0007156 | 3.20 x10^-19^ |
| calcium-dependent cell-cell adhesion | GO:0016339 | 3.20 x10^-19^ |
| cell-cell adhesion | GO:0016337 | 9.52 x10^-13^ |
| synaptogenesis | GO:0007416 | 8.64x10^-12^ |
| synapse organization and biogenesis | GO:0050808 | 2.33x10^-10^ |
| cell adhesion | GO:0007155 | 2.14x10^-07^ |
| extracellular structure organization and biogenesis | GO:0043062 | 3.85x10^-07^ |
| sensory perception of taste | GO:0050909 | 2.26x10^-05^ |
| nervous system development | GO:0007399 | 5.59x10^-04^ |
| peptidyl-tyrosine phosphorylation | GO:0018108 | 8.20x10^-04^ |
| peptidyl-tyrosine modification | GO:0018212 | 9.42x10^-04^ |
| regulation of protein amino acid phosphorylation | GO:0001932 | 1.66x10^-03^ |
| organic cation transport | GO:0015695 | 1.69x10^-03^ |
| regulation of amino acid metabolic process | GO:0006521 | 1.86x10^-03^ |
| synaptic transmission | GO:0007268 | 2.51x10^-03^ |
| chromatin assembly | GO:0031497 | 2.80x10^-03^ |
| cell surface receptor linked signal transduction | GO:0007166 | 5.18x10^-03^ |
| cell-cell signaling | GO:0007267 | 5.79x10^-03^ |
| transmission of nerve impulse | GO:0019226 | 5.79x10^-03^ |
| regulation of phosphorylation | GO:0042325 | 5.79x10^-03^ |
| anatomical structure development | GO:0048856 | 5.79x10^-03^ |
| regulation of phosphate metabolic process | GO:0019220 | 8.39x10^-03^ |
| regulation of phosphorus metabolic process | GO:0051174 | 8.39x10^-03^ |
| system development | GO:0048731 | 9.44x10^-03^ |
| neurological process | GO:0050877 | 1.42x10^-02^ |
| protein-DNA complex assembly | GO:0065004 | 1.45x10^-02^ |
| antigen processing and presentation | GO:0019882 | 1.59x10^-02^ |
| cell recognition | GO:0008037 | 2.56x10^-02^ |
| regulation of cell migration | GO:0030334 | 2.69x10^-02^ |
| sulfate transport | GO:0008272 | 3.36x10^-02^ |

**Additional Table 5**. GO terms corresponding to the “biological process” ontology significantly over-represented in the chromosomes found by the functional enrichment test implemented in the FatiGO program [4]. The one-tailed test, as implemented in the program was used to check for significance. Nominal p-values were adjusted for multiple testing using the FDR [3]. GO terms are arranged by p-values.

| **Chromosome** | **GO term** | **GO ID** | **p-value**  **(FDR-adjusted)** |
| --- | --- | --- | --- |
| 1 | epidermis morphogenesis | GO:0048730 | 1.19708E-15 |
|  | keratinocyte differentiation | GO:0030216 | 0.000102006 |
|  | complement activation | GO:0006956 | 0.00010765 |
|  | N-acetylglucosamine catabolic process | GO:0006046 | 0.000350648 |
|  | chitin metabolic process | GO:0006030 | 0.000350648 |
|  | immunoglobulin mediated immune response | GO:0016064 | 0.003376664 |
|  | innate immune response | GO:0045087 | 0.00754939 |
|  | cellular polysaccharide catabolic process | GO:0044247 | 0.04150541 |
|  | regulation of transcription from RNA polymerase II promoter | GO:0006357 | 0.04150541 |
| 2 | sensory perception of smell | GO:0007608 | 0.001452112 |
| 3 | metal ion homeostasis | GO:0055065 | 0.01876914 |
|  | di-, tri-valent inorganic cation homeostasis | GO:0055066 | 0.01876914 |
|  | chemotaxis | GO:0006935 | 0.02320235 |
| 4 | ethanol oxidation | GO:0006069 | 0.000161857 |
|  | sensory perception of smell | GO:0007608 | 0.008623666 |
| 5 | homophilic cell adhesion | GO:0007156 | 1.74449E-19 |
|  | calcium-dependent cell-cell adhesion | GO:0016339 | 1.5053E-09 |
|  | synaptogenesis | GO:0007416 | 0.000265488 |
|  | synaptic transmission | GO:0007268 | 0.008117234 |
|  | sensory perception of smell | GO:0007608 | 0.01482102 |
|  | proline transport | GO:0015824 | 0.03103764 |
| 6 | chromatin assembly | GO:0031497 | 3.23673E-32 |
|  | protein-DNA complex assembly | GO:0065004 | 1.24526E-31 |
|  | antigen processing and presentation of peptide or polysaccharide antigen via MHC class II | GO:0002504 | 5.30596E-12 |
|  | antigen processing and presentation of peptide antigen via MHC class I | GO:0002474 | 0.000149943 |
|  | organic cation transport | GO:0015695 | 0.002271922 |
| 7 | mismatch repair | GO:0006298 | 1.92542E-06 |
| 8 | defense response to bacterium | GO:0042742 | 4.65624E-11 |
|  | defense response to fungus | GO:0050832 | 0.03247092 |
| 9 | response to virus | GO:0009615 | 9.13894E-06 |
| 10 | regulation of liquid surface tension | GO:0050828 | 0.003135437 |
|  | sensory perception of smell | GO:0007608 | 0.004636393 |
| 11 | sensory perception of smell | GO:0007608 | 2.3344E-106 |
|  | G-protein coupled receptor protein signaling pathway | GO:0007186 | 5.1763E-55 |
|  | regulation of nucleobase, nucleoside, nucleotide and nucleic acid metabolic process | GO:0019219 | 1.36624E-08 |
|  | transcription | GO:0006350 | 3.32197E-08 |
|  | RNA biosynthetic process | GO:0032774 | 1.04732E-07 |
|  | collagen catabolic process | GO:0030574 | 0.01602941 |
| 12 | - |  | - |
| 13 | - |  | - |
| 14 | - |  | - |
| 15 | - |  | - |
| 16 | signal transduction | GO:0007165 | 0.01234142 |
|  | sensory perception of smell | GO:0007608 | 0.01715993 |
| 17 | cell organization and biogenesis | GO:0016043 | 0.04708657 |
|  | transport | GO:0006810 | 0.04708657 |
| 18 | homophilic cell adhesion | GO:0007156 | 0.001473762 |
| 19 | regulation of transcription. DNA-dependent | GO:0006355 | 3.19468E-40 |
|  | system development | GO:0048731 | 0.01394962 |
|  | cell organization and biogenesis | GO:0016043 | 0.01731687 |
|  | lipid biosynthetic process | GO:0008610 | 0.03684681 |
| 20 | defense response to bacterium | GO:0042742 | 0.000274324 |
|  | sensory perception | GO:0007600 | 0.02677984 |
| 21 | - |  | - |
| 22 | glutathione biosynthetic process | GO:0006750 | 0.03328672 |
| X | sensory perception of smell | GO:0007608 | 0.007192522 |
|  | mRNA transport | GO:0051028 | 0.007492005 |
| Y | spermatogenesis | GO:0007283 | 1.40092E-15 |
|  | chromatin assembly or disassembly | GO:0006333 | 0.000177629 |
|  | gonadal mesoderm development | GO:0007506 | 0.01017186 |
|  | fertilization (sensu Metazoa) | GO:0007338 | 0.01066051 |

References

1. Al-Shahrour F, Carbonell J, Minguez P, Goetz S, Conesa A, Tarraga J, Medina I, Alloza E, Montaner D, Dopazo J: **Babelomics: advanced functional profiling of transcriptomics, proteomics and genomics experiments**. *Nucleic Acids Res* 2008, **36**(Web Server issue):W341-346.

2. Al-Shahrour F, Arbiza L, Dopazo H, Huerta-Cepas J, Minguez P, Montaner D, Dopazo J: **From genes to functional classes in the study of biological systems**. *BMC Bioinformatics* 2007, **8**:114.

3. Benjamini Y, Yekutieli D: **The control of false discovery rate in multiple testing under dependency**. *Annals of Statistics* 2001, **29**:1165-1188.

4. Al-Shahrour F, Diaz-Uriarte R, Dopazo J: **FatiGO: a web tool for finding significant associations of Gene Ontology terms with groups of genes**. *Bioinformatics* 2004, **20**(4):578-580.
